# Testicular Torsion with Intact Blood Flow: A Point of Care Ultrasound Case-Series 

**DOI:** 10.24908/pocus.v9i1.17201

**Published:** 2024-04-22

**Authors:** Eric Scheier

**Affiliations:** 1 Pediatric Emergency, Kaplan Medical Center Rehovot Israel; 2 Faculty of Medicine, Hebrew University of Jerusalem Israel

**Keywords:** Testicular Torsion, POCUS, Ultrasound, Partial

## Abstract

Studies have demonstrated the high sensitivity and specificity of pediatric emergency department (PED) point of care ultrasound (POCUS) in the evaluation of testicular torsion. Rarely, testicular torsion may present with intact blood flow. Here, we present a case series of four children with testicular torsion confirmed intraoperatively, who had intact blood flow on POCUS. Markers of testicular torsion can include surrounding hydrocele, heterogenous echotexture, absent venous or high resistance arterial flow, or a torsed cord complex. POCUS practitioners should be familiar with these findings, and the presence of any one or more of these findings should prompt urgent urology consultation to avoid missed torsion.

## Introduction

Studies have demonstrated the high sensitivity and specificity of pediatric emergency department (PED) point of care ultrasound (POCUS) in the evaluation of testicular torsion. Studies have also shown aa significantly decreased length of stay for children evaluated with POCUS prior to or without radiology-performed ultrasound. 

Rarely, testicular torsion may present with intact blood flow. Here, we present a case series of four children with testicular torsion confirmed intraoperatively, who had intact blood flow on POCUS. These cases illustrate that the sonographic diagnosis of torsion is not solely based on the presence or absence of testicular blood flow.

## Materials and Methods

This study was performed in an academic hospital PED. The study was approved by the Kaplan Medical Center Institutional Review Board in accordance with the Declaration of Helsinki. Informed consent was not required. All POCUS images in our department are reviewed for quality control by the author – a pediatric emergency physician with seven years of experience using POCUS. The study period was calendar year 2023. During the study period there were 16 cases of surgically-confirmed testicular torsion. All images were acquired by the author, and in each case the POCUS interpretation was testicular torsion.

**Figure 1:**
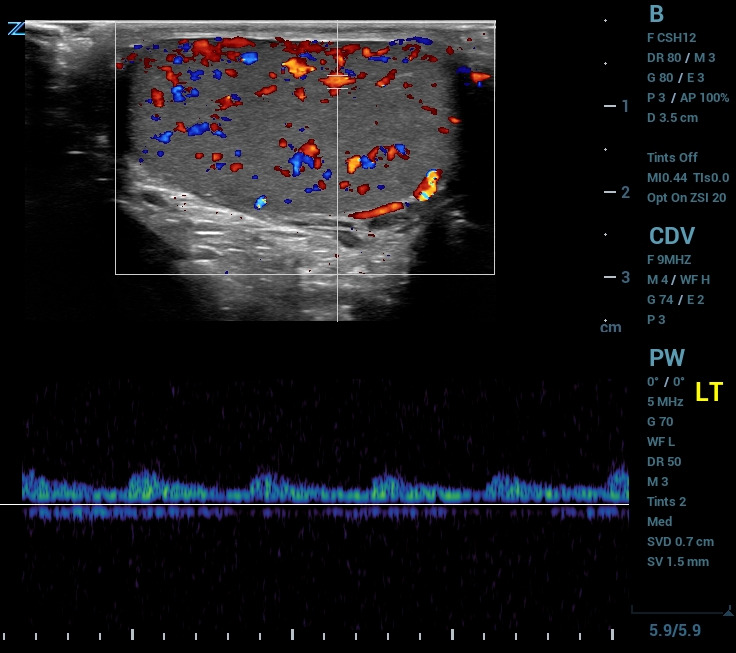
Spectral Doppler image of normal, low-resistance testicular arterial blood flow. Note the homogenous echogenicity and low velocity blood flow that consistently stays above the baseline.

All POCUS images were obtained on a Zonare Z.one ultrasound using a linear high frequency transducer and scrotal presets. Images were obtained on the unaffected side first, both in B-mode and color Doppler, and adequate depth and gain were verified before proceeding to the affected testis. After B-mode and color Doppler imaging were obtained in sagittal orientation on both testes, a “buddy view” or transverse color Doppler image of both testes in the same image was obtained. Spectral Doppler was used in a number of cases (Figure 1). Spectral Doppler is a useful modality to evaluate resistance to forward flow, as discussed further in our cases.

Our facility did not require radiology confirmation of testicular torsion prior to surgery. POCUS was performed at the earliest opportunity, usually in parallel with physical examination. Urology was notified immediately if the POCUS findings were consistent with torsion. The need for surgical exploration was determined by the consulting urologist after examination and either POCUS or radiology-performed ultrasound.

## Case Presentations

### Case 1

**Figure 2:**
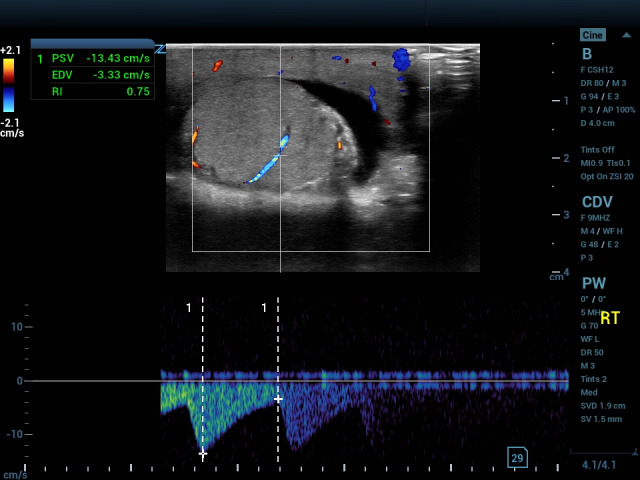
Color Doppler image with linear transducer. the Doppler gate is placed over a blood vessel and shows peaked, high amplitude flow, indicating increased resistance. Hashes are placed at points indicating peak systolic velocity and end diastolic velocity.

A 13-year-old presented with swelling and tenderness of the left testes over the past three days, which worsened overnight. He denied injury, dysuria, vomiting and fever. In triage he reported his pain on the visual analog score (VAS) to be 8. On examination, his left testis was enlarged, hard, and high-riding with an absent cremasteric reflex. POCUS demonstrated a left testis with heterogenous echogenicity and surrounding hydrocele (Video S1) adjacent to the ‘whirlpool sign’, indicating torsed spermatic cord (Video S2). Color Doppler demonstrated scant but central flow with an intratesticular resistive index of 0.75 (normal range 0.48-0.75 in children) (Figure 2) 1. Surgical examination revealed a left testis torsed 720 degrees with adequate color and no sign of ischemia. He underwent bilateral orchiopexy.

### Case 2

**Figure 3:**
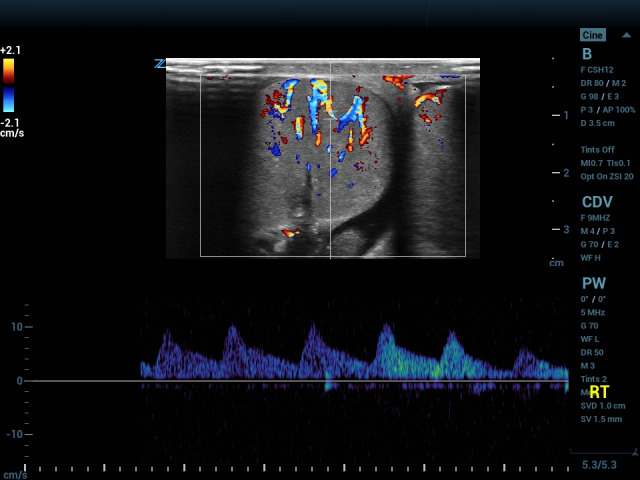
Doppler gate is placed over a blood vessel and shows atardus parvus pattern.

**Figure 4:**
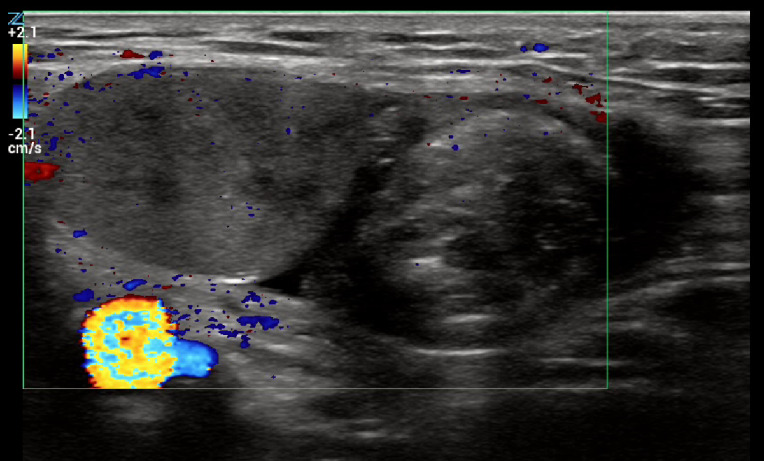
Color Doppler image with linear transducer. The Doppler gate is placed over a blood vessel and shows peaked, high amplitude flow.

A 14-year-old presented with six hours of right scrotal pain. He denied injury, dysuria, vomiting and fever. He had been seen four months earlier due to injury to the right testis. POCUS at that time was unremarkable and he was discharged home. Since then, he had brief episodes of right scrotal pain. He reported a VAS of 7. Examination showed a tender right testis with normal texture and horizontal lie. Cremasteric reflex was absent on the right. POCUS demonstrated heterogenous echotexture with surrounding hydrocele and scant arterial flow, demonstrating a tardus parvus pattern (Figure 3, Video S3). During examination, spontaneous increase in blood flow was seen (Figure 4, Video S4,S5), as was a kinked spermatic cord (Video S6). Given concern for recurrent torsion, bilateral orchiopexy was performed. Surgical examination showed a 90-degree twist.

### Case 3

**Figure 5:**
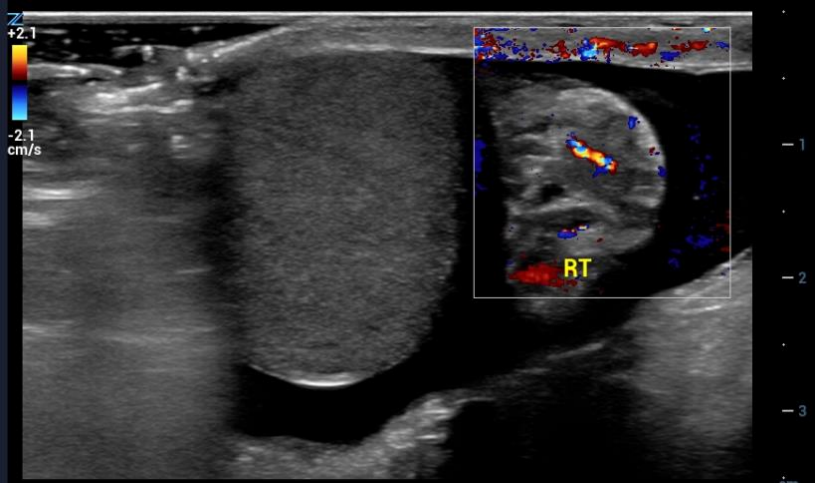
Color Doppler image with linear transducer. The testis is on the left of the color window and the circular cord complex (“whirlpool sign’) is on the right. The cord complex can be difficult to distinguish from epididymis. Here, there is very little flow in the cord complex, as can be seen in torsion. In contrast, epididymitis would present with epididymal swelling and increased blood flow.

A 14-year-old presented with three hours of right inguinal pain that began during exercise. On urination he noted right inguinal swelling and reported a single episode of emesis. His medical history was significant for orchiopexy at one year of age to treat a left undescended testis. Examination showed that his right testis was in the inguinal canal and was tender to palpation. POCUS showed a testis in the inguinal canal adjacent to the cord complex (Figure 5). Color Doppler showed intact central flow (Video S7). Surgical exam revealed a testis that was torsed 180 degrees. Normal color returned with warming, and he underwent right orchiopexy. 

### Case 4

A fifteen-year-old presented with several hours of left testicular pain. He denied emesis and dysuria. Examination showed tenderness to the left epididymis and intact cremasteric reflexes. POCUS was significant for decreased blood flow to the left testis in comparison with the unaffected right testis. There was a small reactive hydrocele adjacent to the left testis (Videos S8,S9). Intraoperatively the left testis was found to be torsed 90 degrees, and the patient underwent orchiopexy. 

## Discussion

Here, we present a case series of testicular torsion with blood flow present to the affected testis on presenting POCUS examination. Physicians using POCUS should be aware that intact blood flow does not rule out testicular torsion.

Testicular torsion is a true surgical emergency and time to detorsion is crucial 2, 3. Absence of venous flow is an early sign of torsion. Absence of venous outflow causes edema to the testes, resulting in swelling and heterogenous echotexture of the testis, and a sterile hydrocele. Edema of the scrotum can be seen as well 4. However, the presence of venous flow in the testis is difficult to record, and its presence or absence does not confirm presence or absence of torsion 5.

Testicular edema causes loss of the mediastinum testis, a hyperechoic stripe traversing the normal testis. Following venous occlusion, arterial flow is decreased. In a case of testicular torsion, arterial flow will be decreased compared with the unaffected testis. This can result in absent or reversed waveforms as edema impedes or reverses forward flow; absence of a dicrotic notch resulting in a monophasic waveform (absence of diastolic flow between systolic peaks); or high amplitude waveforms indicating increasing resistance to flow (Figures 1,2) 6, 7. Conversely, arterial waveform may show a ‘tardus parvus’ pattern with low amplitude and slow upstroke, as seen in stenotic vessels (Figure 3) 8. Resistive index (peak systolic velocity minus end diastolic velocity, with the difference divided by peak systolic velocity) normally ranges from 0.48-0.75 in children, and an elevated resistive index is indicative of compromised perfusion 1, 9. Peripheral flow is not uncommon in testicular torsion and areas of increased echogenicity may be seen secondary to local hemorrhage. Typically, a twist of 450 degrees or more causes complete occlusion of blood flow to the testis 5. Prolonged ischemia will cause marbling of the testis and correlates with poor prognosis 10.

Presence of the ‘whirlpool sign’ indicating a knotted cord complex (Figure 4, Video S2) is diagnostic of torsion, even in the presence of intact testicular blood flow 8. While the ‘pseudomass’ is used to denote the knotted cord, it actually refers to the complex of edematous epididymis, vas deferens, and distal cord vessels that cannot be distinguished sonographically 4. Spermatic cord should not be found adjacent or below the testis, and redundant tortuous cord in the scrotum is abnormal (Video S6) 8. 

This is the first case series of testicular torsion with preserved blood flow on POCUS. Friedman et al. found that POCUS for evaluation of acute scrotum identified testicular torsion with a specificity of 99.1%, and all true-positive cases were identified by POCUS. On image review, they found agreement between POCUS reviewers for all cases of true testicular torsion 11. A subsequent meta-analysis found that POCUS had pooled sensitivity and specificity of 98.4% and 97.2% for testicular torsion 12. Scrotal POCUS can be performed an average of 38-73 minutes before radiology performed ultrasound [11-13], has been shown to decrease PED length of stay by 77 minutes [13], and to decrease time to orchiopexy by over an hour 13. 

However, none of the above POCUS studies address the rare event of torsion with preserved flow. Central testicular blood flow can be seen in an incompletely torsed testicle or in children with thinner spermatic cords that limit the pressure placed on the vessels 8. Testicular pain that resolves suddenly, with normal flow on POCUS, may warrant urology consultation to evaluate for intermittent torsion 14. Continued pain requires urology consultation as well. False-negative ultrasound examinations have been shown to be as high as 41.7% in cases of surgically confirmed testicular torsion 15. Secondary signs on ultrasound that increase the likelihood of testicular torsion include hypoechogenic regions of testis, a surrounding hydrocele, scrotal edema, and a swollen spermatic cord^16^ as well as testicular enlargement, edema, and abnormal axis in comparison with the unaffected side 17. While the ‘whirlpool sign’ is diagnostic of torsion, radiology-performed ultrasound showed poor correlation between the degree of torsion on ultrasound and on surgical exam 18.

## Conclusion

Rarely, testicular torsion may present with preserved blood flow. Markers of torsion can include surrounding hydrocele, heterogenous echotexture, absent venous or high resistance arterial flow, or a torsed cord complex. POCUS practitioners should be familiar with these findings, and the presence of any one or more of these findings should prompt urgent urology consultation to avoid missed torsion.

## Disclosures

The authors received no funding have no financial relationships relevant to this article to disclose. The authors declare no conflict of interest.

## Author Contributions

Dr. Scheier collected the images and wrote the manuscript.

## Supplementary Material

 Video S1 Testis with heterogenous echotexture and surrounding hydrocele.

Video S2 Whirlpool sign representing cord complex is demonstrated in the center of the color window.

Video S3 Testis with heterogenous echotexture and surrounding hydrocele. Scant central blood flow is present on color Doppler imaging.

Video S4 Testis with heterogenous echotexture and surrounding hydrocele. Normal central blood flow is now present on color Doppler imaging.

Video S5 Color Doppler ‘buddy view’ showing two testes with similar echogenicity and blood flow.

Video S6 Color Doppler image. On the left of the color window is the testis. On the bottom right, with linear flow representing blood vessel, is the kinked cord complex.

Video S7 Color Doppler image of testis with heterogenous echotexture and intact central blood flow.

Video S8 Color Doppler image of the left testis with preserved blood flow.

Video S9 Color Doppler buddy view. The right testis is on the left of the screen. The affected left testis located screen right shows decreased blood flow compared to the right (unaffected) testis. Adjacent to the left testis is a small reactive hydrocele.

## References

[R231074930660774] Osemlak P, Jędrzejewski G, Woźniak M, Nachulewicz P (2021). Ultrasound evaluation of long-term outcome in boys operated on due to testicular torsion. Medicine (Baltimore).

[R231074930660771] Gold D D, Lorber A, Levine H (2019). Door to detorsion time determines testicular survival. Urology.

[R231074930660770] Zvizdic Z, Aganovic A, Milisic E, Jonuzi A (2021). Duration of symptoms is the only predictor of testicular salvage following testicular torsion in children: A case-control study. Am J Emerg Med.

[R231074930660782] Sweet D E, Feldman M K, Remer E M (2020). Imaging of the acute scrotum: keys to a rapid diagnosis of acute scrotal disorders. Abdom Radiol (NY).

[R231074930660780] Gupta A, Croake A, Rubens D, Dogra V (2022). Do Not Get It Twisted: Common and Uncommon Manifestations of Testicular Torsion. J Ultrasound Med.

[R231074930660778] Fenton L Z, Karakas S P, Baskin L, Campbell J B (2016). Sonography of pediatric blunt scrotal trauma: what the pediatric urologist wants to know. Pediatr Radiol.

[R231074930660772] Dogra V S, Rubens D J, Gottlieb R H, Bhatt S (2004). Torsion and beyond: new twists in spectral Doppler evaluation of the scrotum. J Ultrasound Med.

[R231074930660777] Gupta A, Croake A, Rubens D, Dogra V (2022). Do Not Get It Twisted: Common and Uncommon Manifestations of Testicular Torsion. J Ultrasound Med.

[R231074930660773] Bandarkar A N, Blask A R (2018). Testicular torsion with preserved flow: key sonographic features and value-added approach to diagnosis. Pediatr Radiol.

[R231074930660776] Shields L B, Daniels M W, Peppas D S, Rosenberg E (2022). Sonography Findings Predict Testicular Viability in Pediatric Patients With Testicular Torsion. Cureus.

[R231074930660765] Friedman N, Pancer Z, Savic R, Tseng F (2019). Accuracy of point-of-care ultrasound by pediatric emergency physicians for testicular torsion. J Pediatr Urol.

[R231074930660768] Mori T, Ihara T, Nomura O (2023). Diagnostic accuracy of point-of-care ultrasound for paediatric testicular torsion: a systematic review and meta-analysis. Emerg Med J.

[R231074930660779] Park J S, Kim D, Chun M K, Choi S J (2009). Implementing Point-of-Care Ultrasound for Acute Scrotal Pain in the Pediatric Emergency Department: Screening Testicular Torsion and Patient Flow Analysis. J Ultrasound Med.

[R231074930660775] Koppel J H, Patt Y S, Berant R (2023). Point-of-Care Ultrasound for the Diagnosis of Pediatric Testicular Torsion: A Retrospective Case Series Analysis. Pediatr Emerg Care.

[R231074930660769] Lacy A, Smith A, Koyfman A, Long B (2023). High risk and low prevalence diseases: Testicular torsion. Am J Emerg Med.

[R231074930660767] Lukosiute-Urboniene A, Nekrosius D, Dekeryte I, Kilda A (2023). Clinical risk factors for testicular torsion and a warning against falsely reassuring ultrasound scans: a 10-year single-centre experience. Emerg Med J.

[R231074930660781] Hosokawa T, Tanami Y, Sato Y, Oguma E (2001). Point-of-care ultrasonography for the diagnosis and manual detorsion of testicular torsion. J Med Ultrason.

[R231074930660766] Hosokawa T, Takahashi H, Tanami Y (2020). Diagnostic accuracy of ultrasound for the directionality of testicular rotation and the degree of spermatic cord twist in pediatric patients with testicular torsion. J Ultrasound Med.

